# Passive doubly curved structures for determining clamping forces applied to X-ray optic assemblies

**DOI:** 10.1107/S1600577523007579

**Published:** 2023-10-10

**Authors:** Eleanor Victoria Bainbridge, Jonathan David Griffiths, Hiten Patel, Jessica Clunan, Peter Docker

**Affiliations:** a School of Engineering, University of Lincoln, Brayford Way, Brayford Pool, Lincoln LN6 7TS, United Kingdom; b Diamond Light Source, Diamond House, Harwell Science and Innovation Campus, Fermi Ave, Didcot OX11 0DE, United Kingdom; RIKEN SPring-8 Center, Japan

**Keywords:** silicon monochromators, X-ray optics, clamping distortion

## Abstract

Clamping of indirectly cryogenically cooled X-ray optics is required to ensure effective heat transfer between the optic and heat exchanger. However, clamping forces can result in distortion of the optical surface of monochromators and mirror systems, which causes angular distortions of the subsequent beam. As such, there is a need for greater understanding of how these optics are assembled and how this affects their performance throughout their life cycle. In this paper, the potential for non-contact, in-process monitoring of the clamping force both during and after assembly using an additively manufactured passive structure based on a doubly curved hyperbolic paraboloid and designed for application to the first crystal for the I20 monochromator at Diamond Light Source is investigated numerically and experimentally. The performance of the passive structure both pre- and post-cryogenic quenching is characterized experimentally. Laser displacement measurements reveal approximately 9 µm total displacement in the passive structure per 100 N of bolt preload, corresponding to an effective magnification of the preload adjustment of approximately 2.5×.

## Abstract 2

A novel approach to in-process monitoring of clamping forces applied to indirectly cooled X-ray optics through use of an additively manufactured passive structure, based on a doubly curved hyperbolic paraboloid, is detailed. The results presented reveal accurate and repeatable displacement at the tip of the structure under preload, both pre- and post-cryogenic quenching, amplifying the adjustment reaction at the bolt by a factor of approximately 2.5.

## 1. Introduction

The Diamond-II upgrade programme at the UK’s national synchrotron facility, Diamond Light Source, is a co-ordinated programme of development that will require improvements to beamline optics, such as mirrors and diffractive optics, to cope with the anticipated increases in incident powers and power densities (Diamond Light Source Ltd, undated ▸). Typically, X-ray optics are mounted in heat exchangers and indirectly cryogenically cooled to minimize thermal distortion on the optical surface under beamline conditions, for example, as a result of favourable thermal conductivity and thermal expansion coefficient values for silicon at low temperatures (Khosroabadi *et al.*, 2022 ▸). Mounting of X-ray optics in heat exchangers can lead to distortion of the optical surface as a result of the applied clamping forces. At present, optics are typically assembled using calibrated spring arrangements and torque wrenches to set preloads, which rely on the operators’ know-how and experience (Dolbnya *et al.*, 2019 ▸). This can lead to optics which do not perform as predicted due to inconsistences in the assembly methodology, resulting in unwanted distortion of the optical surface (Stimson *et al.*, 2017 ▸). Bainbridge *et al.* (2022 ▸) investigated the effect of clamping forces applied during assembly of the first crystal for the I20 monochromator at Diamond Light Source. Finite-element simulations predicted increased slope errors with increasing clamping force, with uneven tightening of bolts resulting in skewed convex distortion of the optical surface. Distortion of the optical surface of monochromators can impact the orientation of the optical surface, adversely affecting the performance of the optic (González, 2012 ▸). As such, ex situ measurement techniques are used for inspection of X-ray optics and to achieve desired slope errors for synchrotron optical systems, such as iterative correction of clamping-force-induced distortion in monochromators (Alcock *et al.*, 2016 ▸). Recent advances in such ex situ techniques include stitching of Fizeau interferograms to form a composite image of X-ray optics for the identification of defects during fabrication or repolishing of mirrors (Huang *et al.*, 2020 ▸; da Silva *et al.*, 2023 ▸). However, the effectiveness of ex situ metrology is somewhat limited as it cannot account for thermal distortion of optics under beamline conditions (Hu *et al.*, 2022 ▸). The effects of beamline conditions and on distortion of the optical surface have been investigated extensively (Zhang *et al.*, 2013 ▸; Chumakov *et al.*, 2014 ▸; Brumund *et al.*, 2021 ▸).

There is scope for high-sensitivity, ex situ monitoring of clamping forces in X-ray optics using passive structures. Such structures could yield vital information on preloads during and after assembly by remaining part of the optic assembly under beamline conditions. In this paper, the potential for utilization of passive structures for in-process monitoring of the clamping force during assembly of X-ray optics is demonstrated. A novel, additively manufactured, doubly curved hyperbolic paraboloid passive structure, designed for application to the first crystal for the I20 monochromator at Diamond Light Source and capable of producing repeatable, measurable deformation under the application of the clamping force, is validated numerically and experimentally. The performance of the passive structure both pre- and post-cryogenic quenching is characterized experimentally, through non-contact monitoring of linear displacement and vibrational velocity.

## 2. Doubly curved passive structure concept

**Figure 1:**
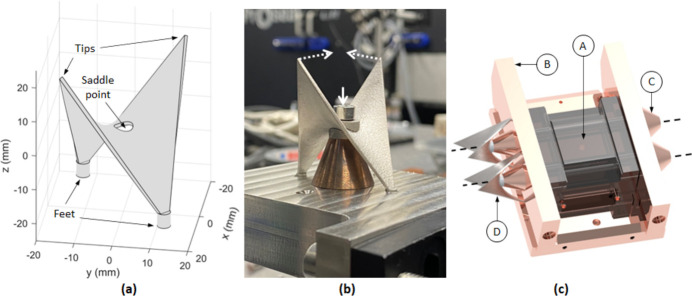
The manufactured passive structure. (*a*) Annotated diagram showing the tips, saddle point and feet. (*b*) Photograph of the passive structure showing adaptors and cone, where the solid white arrow represents the adjustment reaction of the bolt during tightening, with dashed white arrows representing displacement near the tips. (*c*) View of the first crystal for the I20 monochromator at Diamond Light Source: (A) the optical surface of the silicon crystal; (B) copper heat exchanger; (C) copper cones; and (D) passive structures. The dashed black lines represent the through-bolts.

A passive structure based on a doubly curved hyperbolic paraboloid (*z* = *xy*) structure (henceforth referred to as the passive structure) was additively manufactured in stainless steel 316L by direct metal laser sintering (Protolabs Ltd). The passive structure has been designed for application to the first crystal for the I20 monochromator at Diamond Light Source, their flagship X-ray absorption spectroscopy (XAS) and X-ray emission spectroscopy (XES) beamline. The first crystal for the I20 monochromator consists of a silicon crystal, housed in a copper heat exchanger, which has internal channels in the side panels through which liquid nitrogen (LN2) is flowed. The silicon crystal has outer dimensions of 30 mm × 65 mm × 65 mm and is held in the assembly using two stainless-steel through-bolts. The passive structure as well as an envisaged embodiment of the first crystal for the I20 monochromator incorporating the passive structures is shown in Fig. 1 ▸.

The footprint of the passive structure is 29 mm × 29 mm, with a height of 38 mm from the top of the feet to the tip of the structure, as depicted in Fig. 1 ▸(*a*). The cylindrical feet have a radius of 1.75 mm and a height of 4 mm. Figure 1 ▸(*a*) shows a through-hole for the bolt at the saddle point. When fitted to the monochromator assembly, two adaptors sit either side of the passive structure at the saddle point, as shown in Fig. 1 ▸(*b*). The adaptors are planar on one side and match the curvature of the passive structure on the other side. The two cylindrical feet at the base of the passive structure are designed to slot into holes on the outer surface of the copper heat exchanger side panels. The assembly is mounted on a copper cone, as depicted in Fig. 1 ▸(*c*).

The concept underpinning the passive structure is that, as the saddle point is depressed by the adjustment reaction of the through-bolts during tightening of the assembly, this is effectively magnified in the displacement near the tips. This displacement at the tip could then be monitored using conventional contact-based techniques or remotely, for example using a laser displacement measuring system or capacitive sensors. This will enable monitoring of bolt preload during assembly and throughout the lifetime of the assembly to ensure that clamping forces are appropriate and evenly applied.

**Figure 2:**
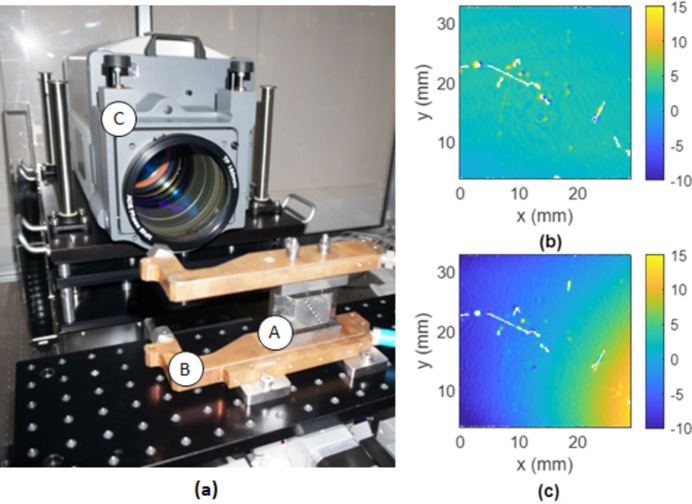
Fizeau interferometry. (*a*) Photograph of the experimental arrangement: (A) stainless-steel replica crystal; (B) copper heat exchanger; and (C) Fizeau interferometer. (*b*) Vertical height profile in nanometres of the surface of the stainless-steel replica crystal for the even tightening case. (*c*) Vertical height profile in nanometres of the surface of the stainless-steel replica crystal for the uneven case.

The importance of ensuring evenly applied clamping forces was demonstrated experimentally. A custom-built Fizeau interferometer stitching system was used to determine the effect of uneven clamping forces being applied to the two stainless-steel through-bolts in a monochromator assembly comprising a copper heat exchange and stainless-steel replica of the first crystal for the I20 monochromator. The system comprised a phase-shifting Fizeau interferometer (Verifire HDX, Zygo Corporation), mounted on an adjustable base, as detailed by da Silva *et al.* (2023 ▸). A photograph of the experimental set-up is shown in Fig. 2 ▸(*a*). Two clamping methodologies were considered: (i) even tightening, in which each bolt was incrementally tightened by one rotation in turn until fully tightened (*i.e.* three rotations), and (ii) uneven tightening, in which one bolt was undertightened to two rotations and the other was overtightened to four rotations, sequentially. The vertical height profiles of the surface of the stainless-steel replica crystal for the even and uneven clamping methodologies are shown in Figs. 2 ▸(*b*) and 2 ▸(*c*), respectively.

Figure 2(*b*) ▸ reveals a largely uniform surface for the even tightening case, whereas a significant amount of distortion is present for the uneven tightening case. This highlights the importance of monitoring clamping forces during assembly of X-ray optics to ensure they are evenly applied.

### 2.1. Finite-element model development

**Figure 3:**
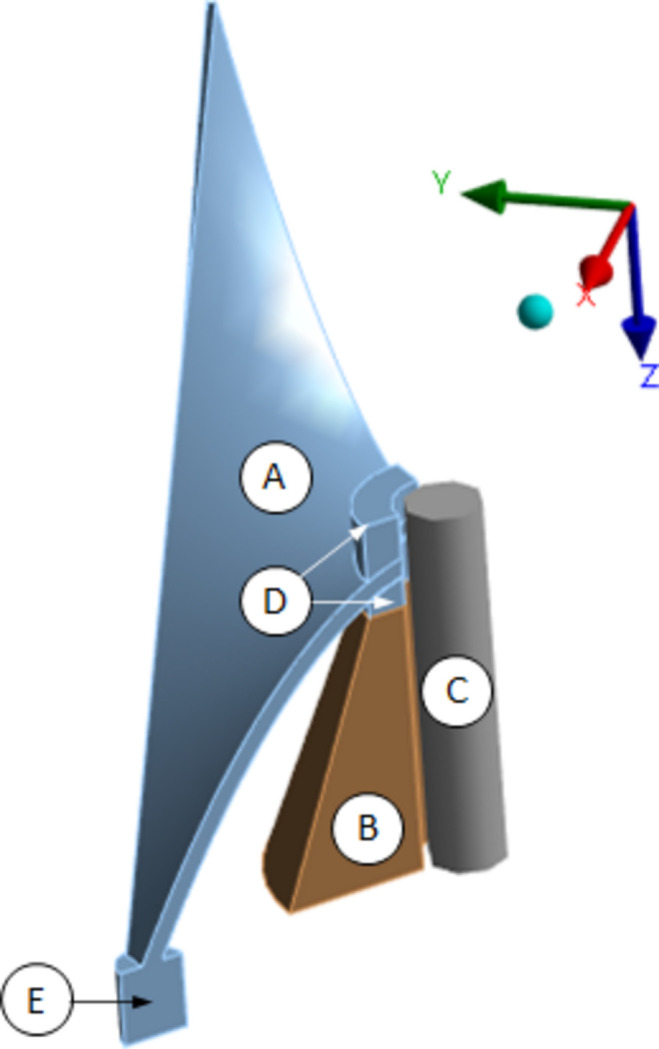
Geometry of the quarter symmetry finite-element model of the passive structure. (A) The passive structure. (B) Copper cone. (C) Bolt. (D) Fasteners. (E) Foot.

A quarter symmetry static structural finite-element model of the passive structure was created using *ANSYS 2023 R1* (https://www.ansys.com/) and used to predict the effect of bolt preload on displacement at the tips, as depicted in Fig. 3 ▸. A linear isotropic material model for the passive structure was assumed, with a Young’s modulus of 193 GPa and a Poisson’s ratio of 0.27. Frictionless contact formulation was applied to the contact between the passive structure and bottom fastener, with no separation contact formulation applied to all other interfaces. A fixed support was applied to the cylindrical foot at the base of the passive structure. A frictionless support was applied to the base of the copper cone. The material properties for copper were taken from the in-built *ANSYS* library. A bolt pretension was applied to a rigid circular connector to simulate the bolt. Standard earth gravity was considered, acting in the positive *z*-direction as depicted in Fig. 3 ▸. The finite-element simulation proceeded in three steps. In the first step, gravity was applied to the structure. In the second step, a preload was applied to the bolt. In the final step, the bolt was locked.

A meshing strategy was adopted which ensured high-quality elements, numerical accuracy and high computational efficiency. A global element sizing of 0.6 mm was specified. A contact sizing of 0.1 mm was specified for the frictionless contact formulation between the passive structure and bottom fastener.

## 3. Results and discussion

The experimental and numerical modelling work was focused on (i) experimental validation of the smart washer concept and (ii) determining the effect of cryogenic temperatures on the performance of the smart washer, as detailed in the following subsections.

### 3.1. Experimental validation

**Figure 4:**
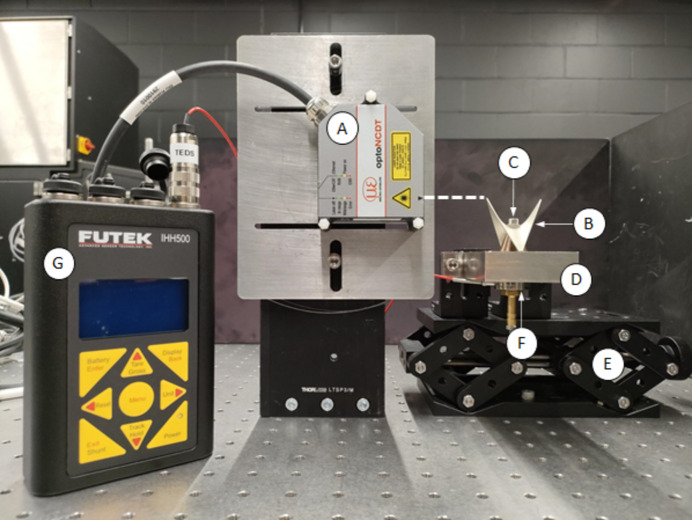
Photograph of the experimental arrangement. (A) Laser displacement measuring system. (B) The passive structure. (C) Through-bolt. (D) Aluminium base plate. (E) Adjustable height stage. (F) Donut load cell. (G) Load cell digital display. The dashed line represents the beam path for the optical displacement measuring system.

The passive structure was mounted on an aluminium base plate using a steel M4 through-bolt. A donut load cell (LTH300, FUTEK Advanced Sensor Technology Inc.) was used to determine the bolt preload, which was monitored using a portable digital display (IHH500, FUTEK Advanced Sensor Technology Inc.). A laser displacement measuring system (ILD2300-20, Opto-NCT) was used to monitor the displacement of the passive structure under load, with a wavelength of 670 nm, pulse duration of <542 µs, measuring rate of 20 kHz and a spot size of 140 µm × 200 µm. The displacement measuring system was operated in diffuse reflection with a resolution of 0.3 µm. The passive structure was mounted on an adjustable height stage, which allowed for the measurement to be taken at a point near the tips. The experimental set-up is depicted in Fig. 4 ▸.

**Figure 5:**
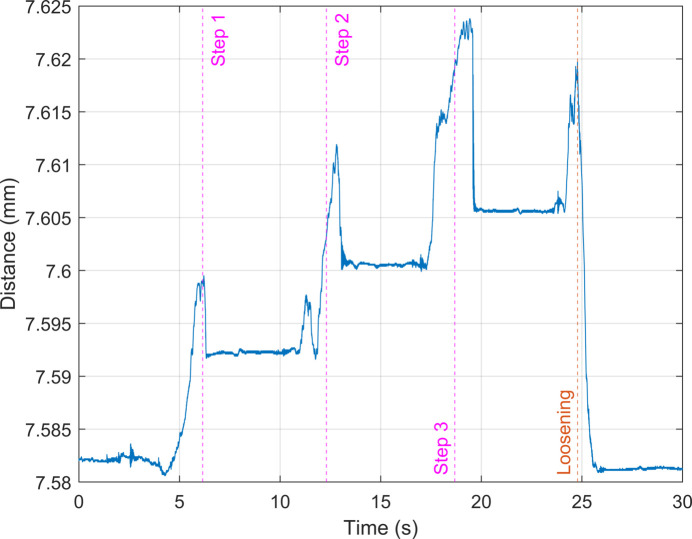
Laser displacement at the tip of the of passive structure over a three-step, incremental tightening process to a preload of approximately 300 N.

The bolt was tightened incrementally. The preload was monitored, with a target preload of 300 N, which represented the upper limit of the load cell. The displacement of the passive structure under load for a three-step tightening process is shown in Fig. 5 ▸.

**Figure 6:**
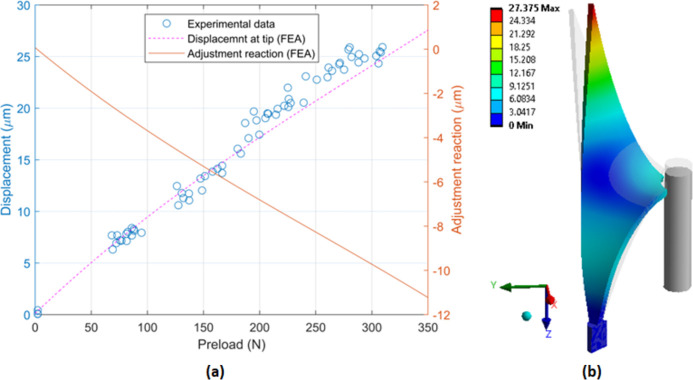
Experimental and numerical modelling results of the passive structure tightening process. (*a*) Optical displacement measurements and predictions for directional displacement at the tip, and adjustment reaction from the finite-element simulation (FEA). (*b*) Total displacement at the tip of the passive structure under load in micrometers for the quarter symmetry finite-element model, showing the undeformed passive structure in grey (300 N preload, 152× scale factor).

Figure 5 ▸ reveals a short recovery period after each increment, consistent with experimental observations from similar experimental arrangements (Cabrera *et al.*, 2021 ▸). After a period of seconds, the displacement of the passive structure settles to a residual value. The displacement is fully recovered upon loosening. The bolt tightening approach was conducted 15 times. Each tightening involved either four or five increments to reach the target preload of 300 N. After each increment, the displacement of the passive structure was allowed to settle to a residual value and the displacement and preload were recorded. The results from the experiments and finite-element simulation are shown in Fig. 6 ▸.

Finite-element predictions reveal a positive cubic relationship between preload and displacement near the tip of the passive structure and show good agreement with the experimental results, as shown in Fig. 6 ▸(*a*). Regarding displacement at the tips, it should be noted that they are translated in two directions under load (that is, horizontally and vertically), as depicted in Fig. 6 ▸(*b*). From Fig. 6 ▸(*a*), it is observed that a 2.5× amplification of the 10 µm adjustment reaction is present in the 25 µm displacement at the tips of the passive structure at a preload of 300 N. This displacement could be monitored remotely during assembly to ensure that, in the case of the first crystal for the I20 monochromator, an even preload was applied to both of the through-bolts depicted in Fig. 1 ▸(*b*).

### 3.2. Effect of cryogenic quenching on performance

**Figure 7:**
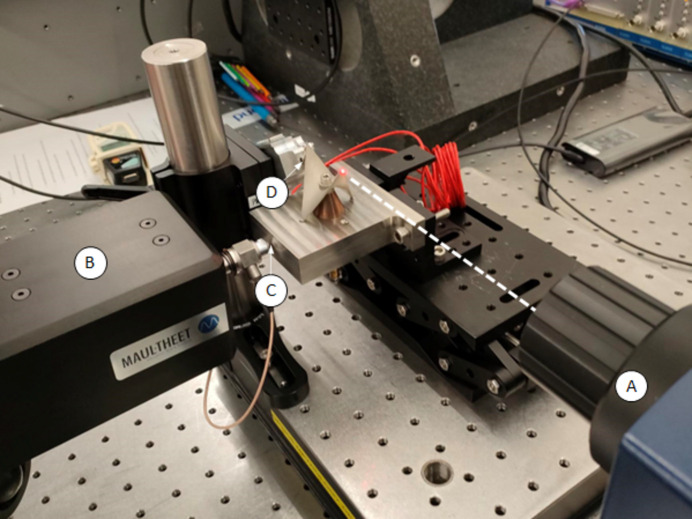
Photograph of the experimental arrangement at the Precision Metrology Laboratory. (A) Laser vibrometer. (B) Automatic modal-impulse hammer. (C) Location of impact on aluminium base plate. (D) Location of capacitive sensor. The dashed line represents the beam path for the laser vibrometer.

The effect of cryogenic temperatures on the performance of the passive structure was determined experimentally at the Precision Metrology Laboratory at Diamond Light Source. The purpose of this was to ascertain the likelihood of failure of the component when reduced to cryogenic temperatures. The performance of the passive structure was characterized before and after rapid quenching in LN2. A He–Ne laser doppler vibrometer (OFV-505, OFV-SR lens, Polytec Inc.) was used to determine the frequency shift as the bolt was tightened, with the measurement taken at a point near the tip of the passive structure at a measuring range and an acquisition frequency of 100 mm s^−1^ V^−1^ and 24 kHz, respectively. A high-pass filter with a cut-off of 2 kHz was applied to filter out low-frequency noise. Data from the laser vibrometer was acquired and analysed using *VibSoft* software (Vibsoft-VL, Polytec Inc.). Excitation of the assembly was provided by an automatic modal-impulse hammer (vImpact-2003, MAUL-THEET GmbH), which was aligned to impact the aluminium base plate with a force of approximately 87 N. Repeatability measurements involved using a non-contact capacitive sensor (CSE1, Micro-Epsilon Ltd) with a controller (capaNCDT 6500, Micro-Epsilon Ltd) to record linear displacement continuously, with an acquisition rate of 1 Hz (averaged down from an internal rate of 2083 Hz). The non-contact capacitive sensor had a working range of 1 mm and an active sensor area of 8 mm. Measurements were taken close to the underside of the tip of the passive structure as shown in Fig. 7 ▸. Bolt preload was monitored using the donut load cell and portable digital display detailed in Section 3.1. A photograph of the experimental set-up is shown in Fig. 7 ▸.

**Figure 8:**
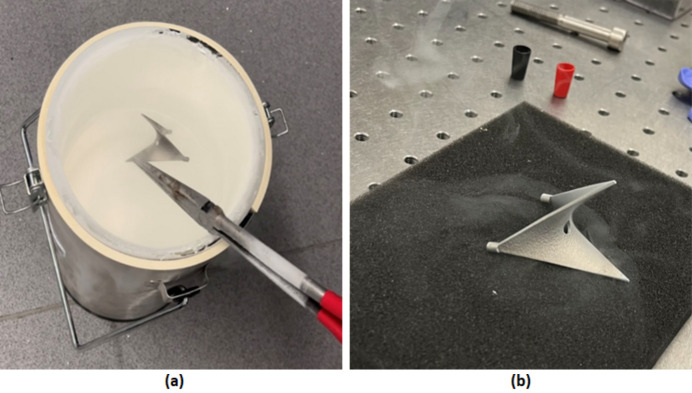
Photographs of the cryogenic quenching process. (*a*) Submersion in LN2. (*b*) Resting at room temperature post-quenching.

The passive structure was cryogenically quenched by submersion in LN2 for a period of approximately one minute. The passive structure was subsequently removed from the LN2 and allowed to return to room temperature before being returned to the experimental set-up. With regard to potential failure of the component, this quenching process effectively represented a ‘worst case’ scenario; under beamline conditions, the transition to cryogenic temperatures would be more gradual. Photographs of the quenching process are shown in Fig. 8 ▸.

**Figure 9:**
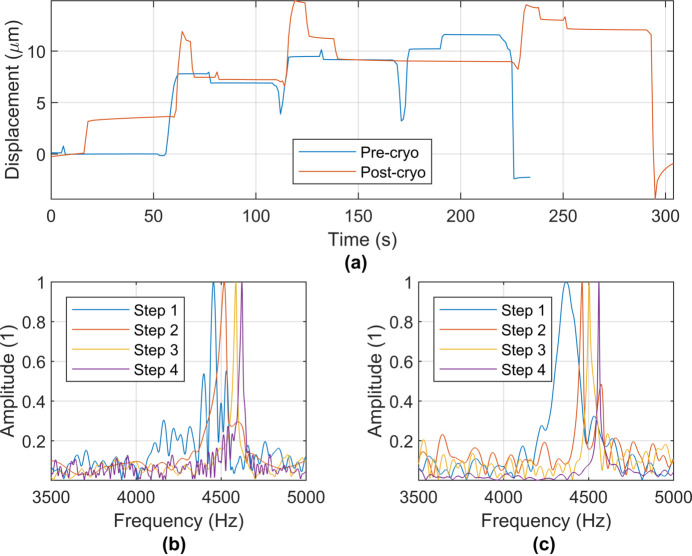
Example traces from pre- and post-cryogenic quenching. (*a*) Capacitive sensor data. (*b*) FFT data from the laser vibrometer pre-cryogenic quenching. (*c*) FFT data from the laser vibrometer post-cryogenic quenching.

For testing, the bolt was tightened incrementally, and the preload monitored, with a target preload of 300 N, as detailed in Section 3.1. This process was repeated five times for both pre- and post-cryogenic quenching. Example traces from the capacitive sensor and fast Fourier transform (FFT) data from the laser vibrometer for pre- and post-cryogenic quenching are shown in Fig. 9 ▸.

Figure 9 ▸ (*a*) reveals consistent deformation both pre- and post-cryogenic quenching at the target preload of 300 N. A frequency shift as the bolt is tightened is shown in Figs. 9 ▸(*b*) and 9(*c*), for pre- and post-cryogenic quenching, respectively. Prior to quenching, the average frequency shift from untight­ened to tightened was 163 Hz (σ = 0.005 kHz), which is comparable with the average frequency shift post-cryogenic quenching of 172 Hz (σ = 0.020 kHz). Whilst this slight discrepancy could be attributed to disturbance of the experimental arrangement during re-mounting of the passive structure, it should be noted that the increased standard deviation post-quenching and slight reduction in frequency observed in Fig. 9 ▸(*c*) relative to Fig. 9 ▸(*b*) are indicative of phase change in the material.

## 4. Conclusions

In this paper, an additively manufactured, passive stainless-steel structure (passive structure) based on a doubly curved hyperbolic paraboloid and intended for use in the in-process monitoring of clamping force during assembly of X-ray optics was detailed. Such a structure could address the inaccuracies associated with conventional clamping methodologies (*i.e.* calibrated spring arrangements) and also remain in place throughout the lifetime of the assembly. The latter is significant, as the effects of improper clamping may be exacerbated under beamline conditions, and therefore could result in poorer performance of the optic during operation than indicated by *ex situ* metrology. Experimentally validated finite-element simulations of the passive structure revealed accurate and repeatable total displacement at the tip of the structure of approximately 9 µm per 100 N preload during tightening, effectively amplifying the adjustment reaction at the saddle point of the structure by a factor of approximately 2.5×. The effect of cryogenic quenching on the performance of the passive structure was determined experimentally at the Precision Metrology Laboratory at Diamond Light Source. The performance of the passive structure was characterized before and after rapid quenching in LN2 using a capacitive sensor laser vibrometer to monitor the effect of bolt tightening on deformation and frequency shift, respectively. The passive structure remained functional post-cryogenic quenching, exhibiting a comparable deformation and frequency shift both pre- and post-cryogenic quenching. Further work will be carried out to determine the effect of cryogenic temperatures on the microstructural and mechanical properties of additively manufactured in stainless steel, with a focus on conditions representative of beamline conditions. Work will also be carried out to characterize the performance of the passive structure when applied to the first crystal for the I20 monochromator at Diamond Light Source, under assembly and beamline conditions.
